# Rehabilitation of post-COVID-19 patients

**DOI:** 10.11604/pamj.2020.36.168.23823

**Published:** 2020-07-09

**Authors:** Mouna Asly, Asmaa Hazim

**Affiliations:** 1 Department of Physical Medicine Physique and Rehabilitation, Casablanca, Morocco; 2 Department of Neurology, University of Mohamed VI for health sciences (UM6SS), Casablanca, Morocco

**Keywords:** Rehabilitation- COVID-19- Disabilities

## To the editors of the Pan African Medical Journal

Since the first case of coronavirus disease presented in Wuhan, China on December 31, 2019, the disease had rapidly spread to the rest of the World accounting for high morbidity and mortality. In Morocco, the first COVID-19 case was confirmed in Casablanca on 2 March 2020. Then, cases have increased alarmingly in the last month. On 23^th^ March 2020, the Moroccan health ministry has authorised doctors to start using hydroxychloroquine or chloroquine and azithromycine in treating persons hospitalized with confirmed COVID-19, if no contraindications. As of 26 May 2020, there have been 7,577 confirmed cases, of which 4,881 have recovered and 202 have died. The SARS CoV-2 syndrome may be characterized by mild respiratory diseases or moderate-to-severe pneumonia, which can cause Acute Respiratory Distress Syndrome (ARDS) and multi-organ failure (Cardiac injury, Neurologic effects, Venous thromboembolism, liver, kidney). Rodriguez-Morales *et al*. in a meta-analysis reported that around 32.8% developed ARDS, 20.3% patients required critical care, and 6.2% developed shock. Fatal outcomes were observed in 13.9% patients [1]. Old age, chronic diseases (hypertension, respiratory and cardiovascular diseases, diabetes), smoking history were significantly associated with the severity of the disease [2,3].

Post COVID-19 ARDS can progress to restrictive respiratory failure due to respiratory muscle weakness, and secondary pulmonary fibrosis with impaired diffusion [4,5] associated with physical deconditioning. Both of ARDS and the prolonged hospital stay due to COVID-19, including time spent in an intensive care units lead respiratory, physical, and psychological dysfonction in patients. This puts them at greater risk for developing post-intensive care syndrome (PICS). PICS is defined as new or worsening impairment in physical, cognitive, or mental health status arising after critical illness and persisting beyond discharge from the acute care setting. [6,7]. The presentation of PICS can be varied. The common symptoms include: Physical impairment: neuromuscular weakness, fatigue, decreased mobility, recurrent falls, deconditioning; Psychological impairment: anxious or depressed mood, sexual dysfunction, sleep disturbances; Cognitive issues: memory disturbance, slow mental processing, poor concentration, Recognizing Delirium). The symptoms can last for a few months to many years post recovery. Family members of patients can be also affected similarly [8,9].

Pulmonary, musculoskeletal, neurological, cardiac, psychological sequelae in some COVID-19 survivors, can affect a person´s ability to perform activities of daily living and lead social restrictions. Rehabilitation care will serve as an important link in the continuum of care, especially for severe forms and dependent elderly with chronic diseases. Rehabilitation interventions must be based on each patient´s individual needs. Therefore, after COVID-19 recovery, patients should be assessed for possible or occuring deficiencies to determine the modalities of rehabilitation (hospital or ambulatory care, intervenants, programs) and they should be managed by a multidisciplinary team which includes physical medicine and rehabilitation doctor, psychologist, physiotherapist, occupational therapist and respiratory therapist, with the use of pharmacological and non-pharmacological interventions. Depending on the deficiencies, Rehabilitation program includes [10]: Neuromotor rehabilitation with: Passive mobilization, active exercises and postures to recover or preserve of joint range of motion of lower limbs, shoulder girdle and cervical spine; Muscle strengthening begin with overall muscle strengthening, using cycloergometers. Progressive-intensity analytical and dynamic muscle strengthening strengthening can be combined with functional exercices (bed mobility, sitting out of bed, sitting balance, sit to stand, walking); Progressive verticalization to fight against orthostatism disadaptation, with appropriate venous compression and monitoring blood pressure and pulse.

Respiratory rehabilitation: Breathing exercises aimed to improve breathing control may potentially be proposed, under assessment and monitoring of exercise tolerance. They are effectiveness for increasing tidal volume and reducing psychological consequences (stress, anxiety and depression); Lung secretion clearance should implement, if necessary, using expiratory flow accelerator (EFA) technique; Neuropsychological rehabilitation: can be proposed after assessment to patients with cognitive disorders, related to hypoxic encephalopathy or to encephalic lesions due to coronavirus (stroke, etc.); Speech therapy is proposed in case of swallowing or voice disorders after prolonged intubation or focal brain damage; Occupational therapy: is indicated to elderly, who lost their autonomy and have limitations in carrying out their daily activities. It allows to encourage of independence and to accelerate for return home; Psychological care is offered to patients with psychological disorders: anxiety, depression, post-traumatic stress; Reconditioning for exercise, including cycloergometer exercises and muscular strengthening, will be helpful to prepare for a return to socio-professional activities. Depending on the case, this rehabilitation can be carried out in several organisational modalities: inpatient, outpatient or at home. In the context of the COVID-19 pandemic, the use of remote monitoring and mobile intelligence technologies with wearable devices can made possible to practice intelligent and digital remote rehabilitation. Remote virtual reality exercises can be offered to these patients, the effectiveness and safety of these tools have been proven to be non-inferior to traditional approaches.

## Conclusion

We share the health situation in Morocco during the COVID-19 pandemic and we propose rehabilitation guidelines for patients recovered from COVID-19 and who are still experiencing disabilities.
